# Invasive Mold Infections: Virulence and Pathogenesis of *Mucorales*

**DOI:** 10.1155/2012/349278

**Published:** 2011-11-01

**Authors:** Giulia Morace, Elisa Borghi

**Affiliations:** Department of Public Health, Microbiology, and Virology, Università degli Studi di Milano, Via C. Pascal 36, 20133 Milan, Italy

## Abstract

*Mucorales* have been increasingly reported as cause of invasive fungal infections in immunocompromised subjects, particularly in patients with haematological malignancies or uncontrolled diabetes mellitus and in those under deferoxamine treatment or undergoing dialysis. The disease often leads to a fatal outcome, but the pathogenesis of the infection is still poorly understood as well as the role of specific virulence determinants and the interaction with the host immune system. Members of the order *Mucorales* are responsible of almost all cases of invasive mucormycoses, the majority of the etiological agents belonging to the *Mucoraceae* family. *Mucorales* are able to produce various proteins and metabolic products toxic to animals and humans, but the pathogenic role of these potential virulence factors is unknown. The availability of free iron in plasma and tissues is believed to be crucial for the pathogenesis of these mycoses. Vascular invasion and neurotropism are considered common pathogenic features of invasive mucormycoses.

## 1. Introduction

The *Mucorales*, which is the core group of the traditional *Zygomycota* [1–3], have been recently reclassified into the subphylum *Mucoromycotina* of the *Glomeromycota* phylum of the kingdom Fungi [4]. This new classification does not include *Zygomycota,* because the authors consider the phylum polyphyletic, indeed the name zygomycosis, which encompassed infections caused by members of *Mucorales* and *Entomophthorales*, has become obsolete [4]. The *Mucorales* are characterized by aseptate (coenocytic) hyaline hyphae, sexual reproduction with the formation of zygospores, and asexual reproduction with nonmotile sporangiospores. They are ubiquitous in nature, being found in food, vegetation, and soil [1–3]. The majority of the invasive diseases are caused by genera of the *Mucoraceae *family, and the resulted disease is called mucormycosis [1–3, 5–7]. Transmission occurs by inhalation of aerosolized spores, ingestion of contaminated foodstuffs, or through cutaneous exposure, the latter being the most important mode of acquisition of mucormycosis in immunocompetent hosts [6, 8]. Risk factors for invasive diseases include uncontrolled diabetes mellitus, haematological malignancies, bone marrow and solid organ transplantation, deferoxamine therapy, corticosteroid therapy, or other underlying conditions impairing the immune system [9]. Limited activity of some principal classes of antifungal drugs (i.e., echinocandins and azole derivatives) as well as vascular invasion and neurotropic activity could explain the high mortality seen in mucormycosis [9, 10]. This paper, together with others published in this special issue, reviews the clinical spectrum of and risk factors for mucormycosis with particular emphasis on the role of fungal traits interacting with human host defences. 

## 2. Epidemiology

A few members of the *Mucorales* (Table 1) are able to grow in human tissues causing a wide spectrum of clinical diseases. The entity and severity of the disease depends on the interaction between the fungus and the host immune defences [5, 7, 11]. In their exhaustive review, Roden and coworkers analysed 929 cases of documented infections caused by members of the former *Zygomycota* since 1885 [9]. They found that 19% of patients did not have any underlying disease at time of infection, while diabetes (36% of cases) was the main risk factor for developing the infection among patients with underlying conditions [9]. More recently, 230 cases of infections were collected in 13 European countries between 2005 and 2007 [12]. The majority of patients (53%) had haematological malignancies (44%) and haematopoietic stem-cell transplantation (9%) as underlying conditions, while only 9% of patients presented diabetes mellitus as the main risk factor [12]. *Rhizopus* spp. are the most common causative agents of invasive mucormycosis, *Mucor* spp. and *Lichteimia* (formerly *Absidia*) spp. rank as second and/or third cause [6–9]. Although mucormycosis remains a highly fatal disease, its burden is still low, as well documented by Pagano and coworkers [13]. They were able to demonstrate that mucormycosis affected about 0.1% of 11,802 patients with hematologic malignancies. Among the 346 cases of proven and probable mold infections, only 14 (4%) were caused by members of *Mucorales* [13]. In immune-competent subjects, mucormycosis generally develops as a consequence of traumatic injuries, and the disease commonly involves skin even if possible dissemination from skin to contiguous organs can occur [9, 11].

## 3. The Infection

Mucormycosis can be classified in rhinocerebral, pulmonary and disseminated, abdominal-pelvic and gastric, and cutaneous or chronic subcutaneous diseases. Common features of rhinocerebral, pulmonary, and disseminated diseases include blood vessel invasion, hemorrhagic necrosis, thrombosis, and a rapid fatal outcome. 

Rhinocerebral mucormycosis is more often associated with uncontrolled diabetes mellitus and ketoacidosis than malignancies or deferoxamine therapy. Inhaled spores colonize at first the upper turbinates and paranasal sinuses and cause sinusitis. Depending on the underlying disease, the fungus can rapidly invade the central nervous system, causing symptoms like an altered mental state, progression to coma, and death within a few days [1–3, 5–11]. 

Pulmonary mucormycosis is commonly seen in patients with leukemia, lymphoma, solid organ or bone marrow transplantation, and diabetes but is occasionally reported also in apparently healthy subjects. Disease manifestations vary from a localized nodular lesion to cavitary lesions and dissemination; in the latter case, massive hemoptysis generally occurs. Crude mortality is lower (60%) in cases of isolated lesions than in severe pulmonary (87%) and disseminated (95%) diseases [9]. 

Gastrointestinal disease is a rare manifestation of mucormycosis, and it is mainly associated with malnutrition in presence of predisposing factors, especially in children with amoebic colitis, typhoid, and pellagra [11]. In the most severe cases, the disease can be characterized by ulceration of the mucosa and invasion of blood vessels with subsequent production of necrotic ulcers, this form of the disease is fatal [3, 11].

Cutaneous mucormycosis may be a primary disease following skin barrier break or may occur as a consequence of hematogenous dissemination from other sites, and the outcome of the disease is strictly dependent on the patients' conditions. Primary cutaneous mucormycosis can involve the subcutaneous tissue as well as the fat, muscle and fascial layers [3]. 

## 4. Treatment

Treatment of mucormycosis combines surgical intervention and antifungal therapy. Liposomal amphotericin B is the drug of choice for the therapy of mucormycosis. The *in vitro* susceptibility testing for amphotericin B gives a broad range of values according to the genus and the species. With the exception of posaconazole, the azole derivatives show a limited *in vitro* activity against *Mucorales*, and the echinocandins have a limited activity against these fungi [14]. Studies of *in vitro* combination of posaconazole with amphotericin B showed synergistic effects against hyphae of some species [15]. In addition, combination therapy with liposomal amphotericin B plus caspofungin or posaconazole and posaconazole with colony-stimulating factor has been successfully used in experimental infections [10, 16–18]. In humans, combination therapy (liposomal amphotericin B plus echinocandins or posaconazole with or without iron chelation) has been used as aggressive antifungal treatment following surgical resection of the damaged tissue [19–23]. Patients treated with combination of antifungal drugs had a better survival outcome than those treated with amphotericin B alone [20, 21]. A promising therapeutic approach consists of the use of iron chelation. Although deferoxamine therapy is associated with a high risk to develop mucormycosis [2, 3, 5–7, 9–11, 24], newer iron chelators (deferiprone and deferasirox) have not been associated with increased risk of mucormycosis and have been used as therapeutic agents in cases of experimental mucormycosis [24].

## 5. Virulence Traits and Pathogenesis

According to Casadevall and Pirofski [25]: “*Quantitative and qualitative measures of virulence vary as a function of host factors, microbial factors, environmental factors, social factors and interactions amongst them*”. This concept is especially true if we consider opportunistic microorganisms such as fungi. Macrophages and neutrophils play the major role in immune defence against agents of mucormycosis. Prolonged neutropenia is thus the main risk factor for developing the disease. Moreover, therapeutic interventions (i.e., corticosteroid therapy), that cause functional defects in macrophages and neutrophils, represent additional risk factors for mucormycosis. Diabetes itself can impair the function of neutrophils contributing to the severity of the mucormycosis in patients with ketoacidosis [26]. An important protective factor against mucormycosis is the low concentration of free iron in plasma and tissues. Many of the underlying diseases listed above as predisposing factors for developing mucormycosis share an iron overload as a consequence of iron tissue burden, elevated serum transferring, or increased nontransferrin-bound iron [24]. Iron is essential for *Mucorales* either enhancing their growth and hyphal development *in vitro* or increasing their pathogenicity *in vivo* [27]. Hemodialysis patients under treatment with deferoxamine (DFO), an iron chelator, are particularly at risk for mucormycosis, and Boelaert and coworkers [28] reported a high mortality (89%) in 46 patients who developed severe mucormycosis during DFO treatment. The same group [27, 29] was able to demonstrate that *Mucorales* use DFO as a xeno-siderophore, being capable to detach iron from DFO in a very efficient manner. More recently, other investigators confirmed the importance of iron in the pathogenicity of *Mucorales* by studying the expression of the FTR1 (high-affinity iron permease of *R. oryzae*) gene and its product [30]. The authors were able to demonstrate the effect of gene disruption and gene silencing on *R. oryzae, *which was unable to acquire iron *in vitro* and showed a reduced virulence in mice. Consistently, anti-Ftr1p antibodies protected mice from *R. oryzae *infection [30]. Angioinvasion with subsequent infarction of the surrounding tissue is uniformly present in all cases of severe disseminated mucormycosis [31]. Specific adhesion to endothelial cells and internalization of the fungus by the endothelial cells are important for the pathogenic strategy of *Mucorales* [32]. More recently, Liu and coworkers [33] demonstrated that a novel host receptor (the glucose-regulated protein 78 [GPR78]) facilitates the invasion of human endothelial cells by *Rhizopus oryzae*. This study demonstrated that in the presence of high iron and glucose concentrations, such as in diabetic subjects, there is a direct relationship between an increased expression of GPR78 and an increased damage to endothelial cells in diabetic mice [33]. Involvement of the CNS is common in invasive mucormycosis, *Mucorales* are capable to gain access to the central nervous system (CNS) by local vessels invasion or direct extension from paranasal sinuses [1–3, 5–11]. Another possible mechanism, involving a retrograde extension of the fungi into CNS by means of the nerves, was hypothesized by Frater and coworkers [31]. By evaluating the histologic features of 20 patients with invasive disease, they found a high percentage of perineural invasion. A further fascinating hypothesis concerning the virulence of *Mucorales*, in particular of *Rhizopus* species—the most common etiological agents of disseminated mucormycosis—is a possible involvement of endosymbiotic bacteria in the pathogenesis of the disease [34]. The authors formulate their hypothesis on the basis of the ability of *Rhizopus *species to live with endosymbiotic toxin-producing bacteria [35] and of the existing link between emergence of mucormycosis and the increased drug resistance of Gram-negative bacteria seen in the recent decades. Later on, both the groups of researchers demonstrated that endosymbiotic toxin-producing bacteria were not essential for the pathogenesis of mucormycosis [36, 37]. Other potential virulence factors of *Mucorales* could be proteolytic, lipolytic, and glycosidic enzymes as well as metabolites like alkaloids or mycotoxins as agroclavine. However, their direct involvement in human cases of mucormycosis has been still to be documented [3].

## 6. Diagnosis

**Figure 1:**
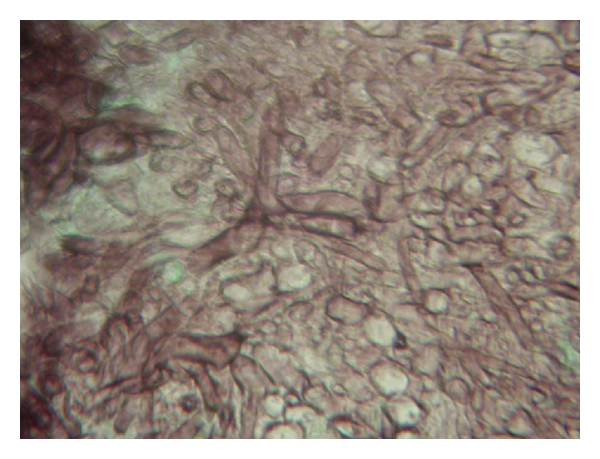
Aseptate hyphae with wide branching angles and large diameter from a lung fungus ball suggestive of mucormycosis (GMS stain 400×).

Histology and culture are still the most important diagnostic approaches for mucormycosis because of the lacking of molecular diagnosis methods standardized or commercially available. Moreover the *β*-1–3 glucan detection is not useful in this kind of infection due to the extremely low content of this molecule in the *Mucorales* [38, 39]. Timely diagnosis of invasive mucormycosis is essential due to the rapid progression of the disease, and because signs and symptoms of the infection could mimic other invasive fungal infections. Tissue biopsies are the clinical specimens of choice and should be submitted to histopathological and microbiological examination. When cultures are performed, it should be remembered that slicing rather than grinding of the samples should be adopted, because grinding could result in the loss of viability due to the coenocytic characteristics of the mycelium. Microscopic detection of aseptate or pauciseptate hyphae with a large diameter and wide branching angles is suggestive of mucormycosis (Figure 1). Histopathological examination of the infected tissues reveals inflammatory response, often entirely filled with neutrophils, invasion of arterial and venous walls (angioinvasion) with subsequent infarction, and perineural invasion [31].

## 7. Conclusion

Invasive mucormycosis is an important cause of morbidity and mortality in patients with impaired immune defence and severe underlying diseases. In immunocompromised or debilitated patients, the disease is rapidly progressive, refractory to antifungal therapy, and often cause of death. Several characteristics of *Mucorales* have been involved in the pathogenesis of the infection as potential virulence factors, but a trait that can be considered a specific determinant of virulence has not been defined yet. Angioinvasion, neurotropism, and iron uptake are common characteristics of *Mucorales* that trigger diseases in humans. Many open issues remain to be clarified on the interaction between members of the *Mucorales *order and the host immune response. Different therapeutic approaches, especially the combination therapy, seem to have a promising impact on the clinical outcome of this infection. However, the development of the most severe forms of mucormycosis and the subsequent outcome is strictly dependent on the efficiency of the host immune system. 

## Figures and Tables

**Table 1:** Agents^a^ of mucormycosis belonging to *Mucorales* order of the *Glomeromycota* phylum.

Order	Family	Genus	Species	Maximum growth temp (°C)
*Mucorales*	*Mucoraceae*	*Rhizopus *	*oryzae*	>37°C
			*microsporus*	>37°C
			*azygosporus*	>37°C
			*schipperae*	>37°C
		*Mucor *	*circinelloides*	>37°C
			*indicus*	>37°C
			*racemosus*	32°C
			*ramosissimus*	36°C
		*Rhizomucor*	*pusillus*	>37°C
		*Lichteimia*	*corymbifera*	>37°C
		*(Absidia)*		
		*Apophysomyces*	*elegans*	>37°C
	*Cunninghamellaceae*	*Cunninghamella*	*bertholletiae*	>37°C
	*Saksenaeaceae*	*Saksenaea*	*vasiformis*	>37°C
	*Syncephalastraceae*	*Syncephalastrum*	*racemosum*	>37°C
